# Chemical Constitution, Pharmacological Effects and the Underlying Mechanism of Atractylenolides: A Review

**DOI:** 10.3390/molecules28103987

**Published:** 2023-05-09

**Authors:** Zhiyi Xie, Minqiu Lin, Xinglishang He, Yingjie Dong, Yigong Chen, Bo Li, Suhong Chen, Guiyuan Lv

**Affiliations:** 1 College of Pharmaceutical Science, Zhejiang Chinese Medical University, Hangzhou 310053, China; 2 Collaborative Innovation Center of Yangtze River Delta Region Green Pharmaceuticals, Zhejiang University of Technology, Huzhou 313200, China; 3 Zhejiang Provincial Key Laboratory of TCM for Innovative R & D and Digital Intelligent Manufacturing of TCM Great Health Products, Huzhou 313200, China

**Keywords:** atractylenolides, *Atractylodes macrocephala*, pharmacological effects, organ-protective

## Abstract

Atractylenolides, comprising atractylenolide I, II, and III, represent the principal bioactive constituents of *Atractylodes macrocephala*, a traditional Chinese medicine. These compounds exhibit a diverse array of pharmacological properties, including anti-inflammatory, anti-cancer, and organ-protective effects, underscoring their potential for future research and development. Recent investigations have demonstrated that the anti-cancer activity of the three atractylenolides can be attributed to their influence on the JAK2/STAT3 signaling pathway. Additionally, the TLR4/NF-κB, PI3K/Akt, and MAPK signaling pathways primarily mediate the anti-inflammatory effects of these compounds. Atractylenolides can protect multiple organs by modulating oxidative stress, attenuating the inflammatory response, activating anti-apoptotic signaling pathways, and inhibiting cell apoptosis. These protective effects extend to the heart, liver, lung, kidney, stomach, intestine, and nervous system. Consequently, atractylenolides may emerge as clinically relevant multi-organ protective agents in the future. Notably, the pharmacological activities of the three atractylenolides differ. Atractylenolide I and III demonstrate potent anti-inflammatory and organ-protective properties, whereas the effects of atractylenolide II are infrequently reported. This review systematically examines the literature on atractylenolides published in recent years, with a primary emphasis on their pharmacological properties, in order to inform future development and application efforts.

## 1. Introduction

*Atractylodes macrocephala*, the dried rhizome of the perennial herb *Atractylodes macrocephala* Koidz. (*Asteraceae* family), possesses warm, bitter, and sweet flavors. Associated with the spleen and stomach meridians, it exhibits various therapeutic effects, such as strengthening the spleen, boosting *qi*, dispelling dampness, arresting perspiration, and stabilizing the fetus [1]. This plant, initially classified as top grade in the *Shen Nong Ben Cao Jing*, is a prominent traditional Chinese medicinal material primarily cultivated in Zhejiang, Gansu, Anhui, Jiangxi, Hunan, and other provinces. Modern pharmacological research has revealed a multitude of benefits of *Atractylodes macrocephala*, including immunomodulation, anti-cancer effects, neuroprotection, spleen fortification, and anti-inflammation [2,3,4,5,6]. Its bioactive constituents consist of volatile oils, polysaccharides, lactones, flavonoids, glycosides, and amino acids, with the pharmacodynamic effects primarily focused on the immune, gastrointestinal, and urinary systems [7]. Among the lactones, atractylenolides (ATLs) are crucial small chemical components contributing to the plant’s efficacy. In recent years, global research on ATLs has expanded, uncovering their remarkable anti-inflammatory, anti-cancer, cardioprotective, and nephroprotective properties. Consequently, this review aims to analyze and synthesize the existing literature on the chemical composition and pharmacological effects of ATLs, enhancing our understanding of their research progress.

## 2. Overview of Atractylenolides

**Figure 1:**
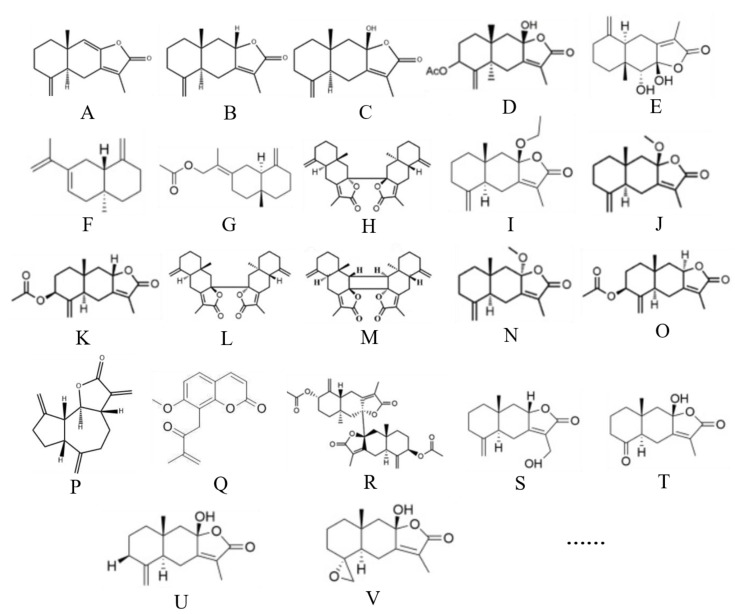
Chemical structures of part of atractylenolides. (**A**) Atractylenolide I, (**B**) atractylenolide II, (**C**) atractylenolide III, (**D**) atractylenolide IV, (**E**) atractylenolide V, (**F**) atractylenolide VI, (**G**) atractylenolide VII, (**H**) biatractylolide, (**I**) 8-β-acetoxyl-atractylenolide III, (**J**) 8-β-methoxy-atractylenolide I, (**K**) 3-β-acetoxyl-atractylenolide I, (**L**) biepiasreorlid, (**M**) biepiasreorlid II, (**N**) 8-α-methoxy-epiasterolid, (**O**) 3-β-acetoxyl-8-epiasterolid, (**P**) dehydrocostus lactone, (**Q**) taraxeryl acetate, (**R**) biatractylolide II, (**S**) 13-hydroxyl-atractylenolide II, (**T**) 4-ketone-atractylenolide III, (**U**) 8-epiatractylenolide III, (**V**) 4(R),15-epoxy-8-β-hydroxyatractylenolide II.

As a sesquiterpene, atractylenolide is one of *Atractylodes macrocephala*’s principal components for exerting its pharmacological actions. Studies have determined some lactone components of atractylenolide, including atractylenolide I, atractylenolide II, atractylenolide III, 8-β-acetoxyl-atractylenolide III, 8-β-methoxy-atractylenolide I, biatractylolide, 8,9-epoxy-atractylenolide, 4,15-epoxy-hydroxyatractylenolide, atractylenolide IV, and hydroxy atractylodes [8,9]. Zhu et al. [7] counted 29 sesquiterpenoids isolated from atractylenolide, including atractylenolide V, atractylenolide VI, atractylenolide VII, 13-hydroxyl-atractylenolide II, 4-ketone-atractylenolide III, 8-epiatractylenolide III, and 4(R),15-epoxy-8-β-hydroxyatractylenolide II. Zhang et al. [10] identified 78 components in the rhizome of *Atractylodes macrocephala* using ultra-performance liquid chromatography and linear ion trap/Orbitrap mass spectrometry, including dehydrocostus lactone, methylatractylenolide II, and other sesquiterpene lactones. From the rhizomes of wild Qimen *Atractylodes macrocephala*, Li et al. [11] identified three lactones: atractylenolide II, taraxeryl acetate, and biatractylolide II, a new dipsesquiterpene lactone. Zhu et al. [12] further studied the rhizomes of wild Qimen *Atractylodes macrocephala* and extracted four sesquiterpenoids, including atractylenolide III and atractylenolide IV. Wang et al. [13] isolated four new sesquiterpene lactones from the rhizome of *Atractylodes macrocephala* containing 3-β-acetoxyl-8-epiasterolid, 8-α-methoxy-epiasterolid, 3-β-acetoxyl-atractylenolide I and biepiasreorlid II, as well as five known sesquiterpene lactones (biatractylolide, atractylenolide IV, biepiasreorlid, 8-β-methoxy-atractylenolide I and 3-β-acetoxyl-atractylenolide I). In addition, Zhang et al. [14] discovered isoatractylenolide I and a new chemical composition 3-β-acetoxyl-atractylenolide I from *Atractylodes macrocephala*. Chemical structures of part of atractylenolides are shown in Figure 1.

**Figure 2:**
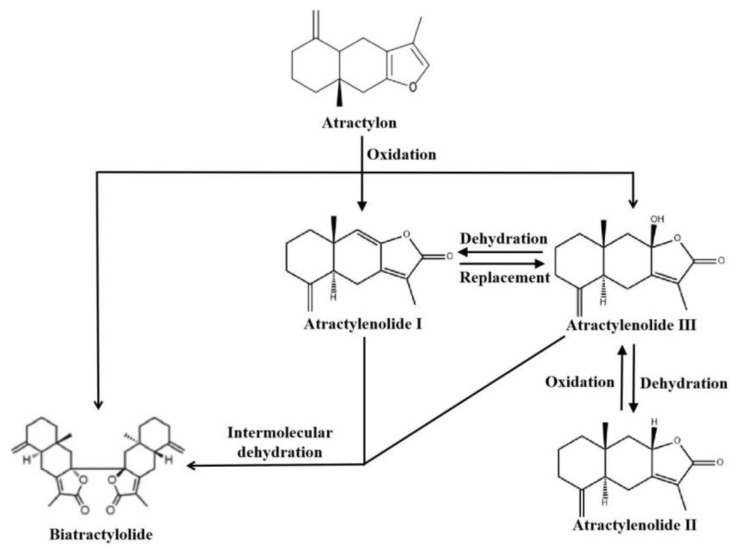
Configuration transformation of atractylenolides. ATLs can be transformed into each other under certain conditions.

Atractylenolide I, II, and III have become the primary focus of research on atractylenolide compounds. These three compounds share the same tricyclic structure [15]. The concentrations of ATL I, II, and III in *Atractylodes macrocephala* vary depending on the plant’s origin and processing methods [16]. Furthermore, interconversions between ATLs have been observed; ATL II can oxidize to ATL III, while ATL III can undergo dehydration to form ATL I [17]. Notably, the hydrogen at carbon 7 in ATL I exhibits high reactivity and can be readily replaced by a hydroxyl group, generating ATL III. However, ATL III is unstable and can undergo intramolecular dehydration, resulting in ATL II. Both ATL III and ATL I can also experience intermolecular dehydration, forming biatractylolide [18]. Other researchers have determined that oxidation of atractylon can produce ATL I, III, and biatractylolide. In the presence of hydrochloric acid–ethanol, ATL III can dehydrate to form ATL II [19,20]. Configuration transformation of atractylenolides is shown in Figure 2.

## 3. Pharmacological Effects of Atractylenolides

### 3.1. Anti-Inflammatory Effects 

Inflammatory disorders are diverse and complex, but from pathophysiology in Chinese medicine point of view, they are nothing more than a battle between good and evil, a yin–yang imbalance, and an ascending and descending disorder of activities of *qi*. *Atractylodes macrocephala* generates fluid and supports righteousness, has a certain therapeutic effect on inflammation. ATL I, III primarily inhibit TLR4/NF-κB, PI3K/Akt, JAK2/STAT3, MAPK, and FPR1/Nrf2 signaling pathways to reduce pro-inflammatory cytokine production and expression of inflammatory factors for the treatment of a variety of inflammatory conditions, but there are fewer reports on the anti-inflammatory effects of ATL II.

#### 3.1.1. Anti-Inflammatory Effects of Atractylenolide I

It was determined that ATL I can reduce lipopolysaccharide-induced release of IL-6 and TNF-α from macrophages RAW264.7, which may have an anti-inflammatory effect by inhibiting ERK1/2, p38, NF-κB signaling pathway [21,22]. More et al. [23] reported that ATL I reduced the inflammatory response of lipopolysaccharide (LPS)-induced inflammatory BV-2 microglia by reducing nuclear translocation of NF-κB and inducing HO-1, as well as ameliorating motor dysfunction, reducing microglia activation, and attenuating dopaminergic neurodegeneration in tetrahydropyridine-poisoned mice. ATL I also protects septic mice from cecum ligation puncture by reducing pro-inflammatory cytokines and LPS levels, as well as improving liver and kidney function [24].

Angiogenesis can cause tumors and many chronic inflammatory diseases. According to Wang et al., ATL I inhibited the secretion of pro-inflammatory cytokines and down-regulated the angiogenic factors VEGF, PlGF, and NO, reducing the in vivo vascular index and in vitro microvascular growth of Freund’s complete adjuvant (FCA) and LPS-induced inflammatory mice for anti-inflammatory purposes [25]. Atherosclerosis is an inflammatory pathology that accompanies the process of vascular damage, ATL I reduces LDL-induced vascular smooth muscle cell proliferation and migration, inflammatory cytokine production and MCP-1 expression, and inhibits p38-MAPK and NF-κB activation, which has beneficial effects on atherosclerosis [26].

Furthermore, in vivo studies revealed that 50 mg/kg ATL I improved the inflammatory response of dextran sulfate sodium salt (DSS)-induced colitis mice via the SPHK1/PI3K/Akt signaling pathway, reduced the expression of inflammatory factors TNF-α, IL-6, and IL-1β, and regulated fructose/galactose-related metabolism by targeting the SPHK1 and B4GAT2 genes, thereby regulating the diversity and abundance of intestinal flora, which is one of the medications that could be used to treat colitis [27].

#### 3.1.2. Anti-Inflammatory Effects of Atractylenolide II

Only one study indicated that ATL II inhibited lipopolysaccharide-induced NO secretion in macrophage RAW264.7 cells [28].

#### 3.1.3. Anti-Inflammatory Effects of Atractylenolide III

In vivo investigations revealed that 100 g/mL ATL III inhibited the release of NO, TNF-α, IL-6, and PGE2 linked with the MAPK/NF-κB signaling pathway, resulting in anti-inflammatory actions [29]. By downregulating pSTAT6 and MDM2 levels, ATL III inhibits thymic stromal lymphopoietin (TSLP)-induced proliferation of HMC-1 human mast cells, reduces pro-inflammatory cytokine production, decreases Bcl2 and proCaspase-3 levels, and increases p53, Caspase-3, and cleaved PARP levels [30]. ATL III regulates oxidative stress via the Nrf2 and FPR1 pathways, reduces the expression of inflammatory markers IL-1 and TNF-α, and alleviates trinitrobrnzen sulfonic acid (TNBS)-induced acute colitis through altering the formation of gut microbiota in a mice model [31]. By activating the AMPK/SIRT1/PGC-1α signaling pathway, ATL III not only eliminated lipopolysaccharide-induced intestinal barrier damage and reduced cellular mitochondrial dysfunction in IEC-6 cells, but also alleviated DSS-induced mitochondrial dysfunction and improved colitis in mice with ulcerative colitis [32].

Asthma is a common inflammatory allergic disease. In the lung tissue of asthmatic mice, ATL III can relieve ovalbumin-induced lung tissue damage, reduce inflammatory cytokine production, balance oxidative stress, and inhibit STAT3 expression [33]. Zhu et al. [34] discovered that ATL III prevented IL-4-induced apoptosis and promoted proliferation in human bronchial epithelial-like cells 16HBE, and inhibited Cleaved Caspase-1, ASC, and NLRP3 expression. Animal studies further demonstrated that ATL III regulates Th1/Th2 balance by reducing ovalbumin-induced activation of NLRP3 inflammatory vesicles in asthmatic mice for asthma therapy. In addition, ATL III inhibited IgE/Ag-induced degranulation of RBL-2H3 in rat basophilic leukemia cells, possibly associated with elevated intracellular Ca^2+^ levels, and also reduced IL-4 and TNF-α production and inhibited phosphorylation of Lyn, Fyn, Syk, LAT, PLCγ, Gab2, Akt, p38, and JNK kinases [35].

The inflammatory response triggered by a spinal cord injury may hinder recovery. ATL III improves the extent of the injury and functional recovery in spinal cord injured rats by promoting the conversion of lipopolysaccharide-induced inflammatory BV2 microglia from M1 to M2 phenotype, attenuates microglia/macrophage activation, and thus inhibits the expression of inflammatory factors, possibly in part by inhibiting the NF-κB, MAPK/JNK, and Akt signaling pathways to produce an anti-spinal cord injury effect [36].

ATL III also has an anti-inflammatory effect on microglial cells. Ela et al. [37] discovered that ATL III downregulated TLR4 levels in immortalized mice microglial MG6 cells, causing inhibition of p38 and JNK, reducing the production of pro-inflammatory cytokines and enzymes in lipopolysaccharide-stimulated microglia. Zhou et al. [38] established MCAO mice model and discovered that ATL III can prevent cerebral ischemia by reducing neuroinflammation caused by JAK2/STAT3/Drp1-mediated mitochondrial fission.

**Figure 3:**
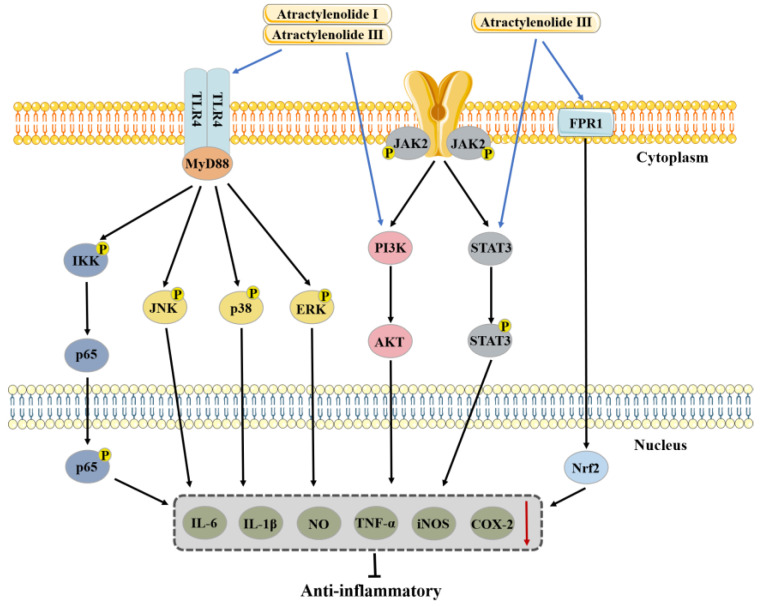
Anti-inflammatory effect and mechanism of atractylenolide I and III. ATL I and III can produce anti-inflammatory effects through TLR4/MyD88 and PI3K/AKT signaling pathways, ATL III can produce anti-inflammatory effects through STAT3 and FPR1/NRF2 signaling pathways.

Anti-inflammatory effect and mechanism of atractylenolide I, II, and III are shown in Figure 3 and Table 1.

### 3.2. Anti-Cancer Effects 

It is recorded in *The Yellow Emperor’s Canon of Internal Medicine* that “When there is sufficient healthy *qi* inside, pathogenic factors have no way to invade the body”. Lack of healthy *qi* is the internal cause of cancer. *Atractylodes macrocephala* supports and tonifies the body, and has a good anti-cancer effect.

#### 3.2.1. Anti-Cancer Effects of Atractylenolide I

ATL I inhibits cancer cell proliferation and induces apoptosis primarily by regulating the PI3K/Akt, JAK2/STAT3, TLR4/MyD88/NF-κB, Notch, and ERK/GSK3β signaling pathways to treat colon, gastric, lung, melanoma, ovarian, breast, bladder, leukemia, prostate, and cervical cancers.

In human colon adenocarcinoma HT-29 cells, ATL I decreases proliferation, accelerated apoptosis, produces DNA fragmentation, and may have anti-colon adenocarcinoma actions through modulating Caspase and Bcl-2 expression in cells [39]. By reducing PDK1 and inhibiting FoxO1 phosphorylation, ATL I can promote the apoptosis of colorectal cancer cells HCT 116 and increase the sensitivity of oxaliplatin [40]. By inhibiting Drp1-mediated mitochondrial division to suppress NLRP3 inflammatory vesicle activation in colitis-associated colorectal cancer, Qin et al. [41] determined that ATL I reduced cell viability and induced apoptosis in human colon cancer cells HCT116 and SW480, and also effectively inhibited colon tumorigenesis in azoxymethane (AOM)/DSS model mice. ATL I decreases glucose metabolism in vitro and in vivo by decreasing the Akt/mTOR signaling pathway, and thereby limiting colorectal cancer cell growth [42]. Tang et al. [43] further demonstrated that ATL I reduces cancer cell stemness and invasiveness by decreasing the activity of colorectal cancer stem cells and restricting the transfer of carcinogenic miR-200c via extracellular vesicle uptake, thereby inhibiting the PI3K/Akt/mTOR signaling pathway. By blocking the JAK2/STAT3 signaling pathway, ATL I also decreases the proliferation and causes apoptosis of human colorectal cancer cells HCT116 and SW480, slows glycolysis in CRC cells by reducing HK2 expression, and inhibits tumor growth in xenograft colorectal cancer mice [44]. On human colon cancer cells HCT116 and mice colon cancer cells MC38, Xu et al. [45] discovered that ATL I could bind to the target protein PSMD4 and enhance immunoproteasome antigen processing activity and MHC-I-mediated antigen presentation, increasing the cytotoxic response of CD8+ T cells and improving tumor response to immunotherapy. Furthermore, ATL I inhibits the production of intestinal adenomas in vitro and in vivo via activating autophagy and inhibiting D-dopachrome intercalating isomerase [46].

ATL I has pharmacological actions against ovarian cancer. It has been demonstrated to suppress CDK1 expression, control cell cycle, and limit proliferation in human ovarian cancer cells via the PI3K/Akt signaling pathway, leading to anti-ovarian cancer actions [47]. Liu et al. [48] determined that ATL I can reduce the expression of TLR4/MD-2 complex on the surface of epithelial ovarian cancer SKOV3 EOC cells, inhibit the MyD88/NF-κB signaling pathway, down-regulate NF-κB, Akt, and IDO1 protein expression, and inhibit the secretion of IL-6, TGF-β1, VEGF, and IL-17A, further reduce the level of regulatory T cells, and attenuate the proliferative response and antitumor cytotoxicity of T lymphocytes exposed to tumor cell supernatants.

By studying the induction of apoptosis in NSCLC and A549 cells, Qian et al. [49] demonstrated that ATL I has significant anti-tumor activity, not only activating ERK1/2 to inhibit SP1 protein levels, but also decreasing Stat3 protein levels, thereby inhibiting PDK1 expression. Liu et al. [50] discovered that ATL I inhibits proliferation and induces apoptosis in human lung cancer A549 and HCC827 cells via the mitochondria-mediated apoptosis pathway.

ATL I has anti-melanoma properties. Yan et al. [51] reported that ATL I activates the ERK/GSK3β signaling pathway in melanoma B16 cells, causing cellular G1 phase arrest and death, and that the GSK3β inhibitor lithium chloride reverses this effect. By blocking the JAK2/STAT3 signaling pathway, ATL I has also been demonstrated to decrease cell viability, induce apoptosis, and impede cell migration in human melanoma cells A375, as well as down-regulate the levels of STAT3 target genes Bcl-xL, MMP-2, and MMP-9 [52].

According to in vitro and in vivo investigations, ATL not only slows cell migration and decreases CTGF release in triple-negative breast cancer cells, but also reduces CTGF expression in fibroblasts and increases the susceptibility of triple-negative breast cancer cells to paclitaxel. The combination of ATL I and paclitaxel improves triple-negative breast cancer treatment [53]. ATL I can also treat breast cancer by down-regulating the expression of TPI1 and GPI [54]. TLR4 and NF-κB are also overexpressed in breast cancer. TLR4 antagonist ATL I inhibits the TLR4/NF-κB signaling pathway in breast cancer cells and reduces NF-κB-regulated cytokines, cell proliferation, migration, invasion, and promotes apoptosis, leading to an anti-breast cancer effect [55].

ATL I also has therapeutic effects on other cancers. It can inhibit gastric cancer cell viability and induce apoptosis through modulating the Notch signaling system while reducing the ability of gastric cancer stem cell spheroid formation to limit gastric cancer stem cell-like cell proliferation [56]. Other research discovered that ATL I causes overexpression of CD14 or CD14/CD68, improves phagocytosis, and induces death and differentiation in human K562 chronic myeloid leukemia (CML), Jurkat T lymphoma cells, and U937 acute myeloid leukemia (AML), which has the potential to develop new leukemia treatments [57]. ATL I also promoted apoptosis in bladder cancer cells T-24, 253J and regulated cell cycle not only by activating the mitochondrial apoptosis pathway, but also by inhibiting the PI3K/Akt/mTOR signaling pathway, according to Yu et al. [58]. In vivo studies also showed that ATL I reduced tumor volume and tumor weight in nude mice with bladder cancer transplant tumors. By inhibiting the expression of Hsp27, ATL I can inhibit the proliferation of prostate carcinoma cells and enhance the efficacy of cabotinib [59]. Han et al. [60] determined that ATL I decreased the proliferation of human cervical cancer cells Hela and SiHa, and that combination treatment with the P2X7R receptor antagonist JNJ increased cytostatic effect on SiHa cells.

#### 3.2.2. Anti-Cancer Effects of Atractylenolide II

ATL II inhibits cancer cell proliferation and causes apoptosis primarily through regulating the PI3K/Akt, JAK2/STAT3, Ras/ERK, JNK/ERK-Nrf2-ARE, and LncRNA XIST/miR-30a-5p/ROR1 signaling pathways to treat colon, gastric, lung, melanoma, breast, and prostate cancers.

ATL II decreased the proliferation and viability of human colorectal cancer cells by disrupting the LncRNA XIST/miR-30a-5p/ROR1 signaling pathway, reducing cancer cells’ chemosensitivity and improving the efficacy of chemotherapy [61]. By inhibiting the Ras/ERK and PI3K/Akt signaling pathways, ATL II reduced the proliferation and motility of HGC-27 and AGS cells, and triggered apoptosis in human gastric cancer cells by up-regulating Bax and down-regulating Bcl-2 expression [62]. ATL II inhibits the cell cycle of melanoma B16 cells in the G1 phase and promotes apoptosis by activating p38 and inactivating ERK and Akt. The use of PFTα, a chemical inhibitor of p53, significantly reduces ATL II-mediated growth inhibition and apoptosis, revealing that ATL II’s apoptosis is perhaps dependent on p53 [63]. Fu et al. [64] discovered that ATL II can inhibit the expression of p-STAT3, p-Src, Mcl-1, and Bcl-xL in cancer cells, while overexpression of STAT3C, a constitutively active variant of STAT3, reduced the effect of ATL II, implying that it may inhibit STAT3 signaling to produce an anti-melanoma. ATL I and II have also been demonstrated to decrease cell migration and stimulate B16 differentiation in melanoma cells by suppressing the Ras/ERK and PI3K/Akt signaling pathways [65]. By blocking M2-like polarization, Zhang et al. [66] discovered that ATL II decreased the metastatic capacity of TAMs-induced lung cancer cells A549 cells. Through the JNK/ERK-Nrf2-ARE signaling pathway, ATL II inhibits 17β-estradiol-induced malignant transformation of human mammary epithelial MCF 10A cells and reduces N-nitroso-N-methylurea-induced tumorigenesis in rat mammary tissue, and leads to the increased expression of Nrf2, nuclear translocation, and downstream detoxification enzymes [67]. ATL II also suppresses the proliferation and induces death of human prostate cancer cells DU145 and LNCaP, and arrests the cell cycle in the G2/M phase through mechanisms that possibly relate to AR inhibition, PIAS1 activation, and inhibition of the JAK2/STAT3 signaling pathway [68].

#### 3.2.3. Anti-Cancer Effects of Atractylenolide III

There is relatively little research on the anticancer activity of ATL III, which mainly inhibits cancer cell proliferation and promotes apoptosis primarily through the JAK3/STAT3 and miR-195-5p/FGFR1 signaling pathways to treat colon, gastric, lung, and liver cancers.

By promoting Bax, Caspase-9, and Caspase-3, inhibiting Bcl-2 protein expression, and regulating the Bax/Bcl-2 apoptotic signaling pathway in human colon cancer cells, ATL III inhibits cell proliferation and induces apoptosis in HCT-116 cells, producing anti-colon cancer effects [69]. Kang et al. [70] determined that ATL III reduces the viability of human lung cancer A549 cells, increases LDH release, induces apoptosis, and has anti-lung cancer effects, possibly by increasing Caspase-3 and Caspase-9 protein expression, activating the mitochondrial pathway, leading to PAPR cleavage. By upregulating miR-195-5p and downregulating FGFR1 expression, ATL III suppresses proliferation and promotes apoptosis in human hepatoma cells HepG2 and SMMC7721 [71]. Liu et al. [72] discovered that ATL III activates anti-tumor immunity by inhibiting the phosphorylation of Jak3, Stat3, and the nuclear translocation of Stat3, and downregulating the expression of IDO in interferon-γ-induced lung cancer cells, and its interaction site with Jak3 protein is leucine 905. It was also discovered that ATL III combined with docetaxel can reduce the proliferation and induce apoptosis of gastric cancer cells AGS and SGC-7901, which may occur partially through inhibiting the expression of FGFR1, FGFR2, and FGFR4 [73]. In addition, ATL III can also reduce precancerous lesions and inhibit angiogenesis in rats by inhibiting the expression of HIF-1α and VEGF-A, and down-regulating the expression of DLL4 [74].

**Figure 4:**
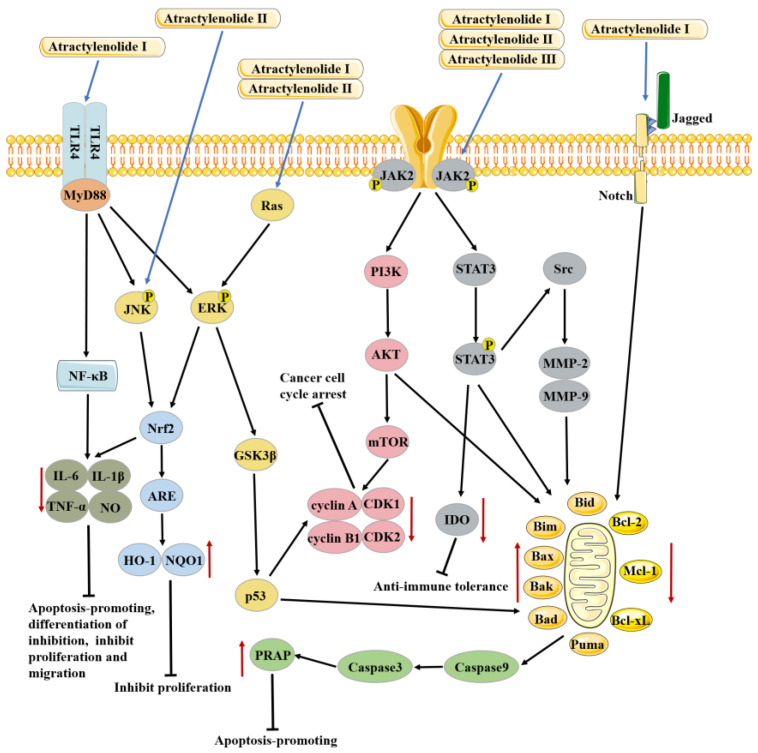
Anti-cancer effects and mechanism of atractylenolide I, II, and III. ATL I can produce anti-cancer effects through TLR4 / MYD88, Ras/ERK, JAK2 and Notch signaling pathways, ATL II can produce anti-cancer effects through JNK, Ras/ERK and JAK2 signaling pathways, ATL III can produce anti-cancer effects through JAK2 signaling pathway.

Anti-cancer effects and mechanism of atractylenolide I, II, and III are shown in Figure 4 and Table 2.

### 3.3. Organ-Protective Effects

#### 3.3.1. Protection of the Gastrointestinal Mucosa

*Atractylodes macrocephala* has the effect to tonifying the spleen and stomach, and the *Materia Medica* states that “Of all the traditional Chinese medicines for invigorating the spleen and stomach, no one is better than *Atractylodes macrocephala*”, and it has a protective impact on gastrointestinal mucosa damage. TLR4/MyD88/NF-κB, SCF/C-kit, and AMPK signaling pathways are primarily inhibited by ATL I and III, which provide protection to the gastrointestinal mucosa, but there are no studies on the protects the gastrointestinal mucosa of ATL II.

Researchers determined that ATL III inhibited 6% ethanol-induced PRGM cell death and cell membrane damage in rat gastric mucosa, and ATL III may have an anti-gastric ulcer effect by inhibiting MMP-2 and MMP-9 in a rat model of gastric ulcer induced by 70% ethanol [75]. Diabetic gastroparesis (DGP) is a common diabetes complication. ATL I increases STZ-induced gastric ICC proliferation, reduces stomach oxidative stress and restores gastric function in DGP rats by activating the SCF/C-kit signaling pathway [76].

Furthermore, ATL I, III can protect the mucosa of the intestine. ATL I has been shown to promote IEC-6 migration and proliferation in intestinal epithelial cells, increase polyamine content, cytoplasmic free Ca^2+^ concentration, and TRPC1 and PLC-γ1 expression, and reverse the effects of DL-α-difluoromethylornithine [77]. ATL I prevents antibiotic-induced dysbiosis of intestinal flora in mice and decreases LPS and IL-1β levels by modulating the TLR4/MyD88/NF-κB signaling pathway [78]. In addition, ATL I can also relieve oxidative stress-induced glucose metabolism dysfunction in colon mucosal epithelial cells by inhibiting the expression of miR-34a-5p, which can effectively prevent irritable bowel syndrome [79]. Huang et al. [80] discovered that ATL III inhibited TGF-β1-induced IEC-6 cell invasion and migration by activating the AMPK signaling pathway, effectively preventing the EMT process and providing a new avenue for medication development of intestinal fibrosis drugs.

#### 3.3.2. Cardiac and Renal Protective Effects

*Atractylodes macrocephala* is good at strengthening the spleen and stomach, as well as tonifying the heart and kidney. It is written that “*Atractylodes macrocephala* can be helpful for nourishing the heart when used with calming medicines; be helpful for nourishing the kidneys when used with nourishing medicines. It is the key medicine to nourish the body, so it can benefit the kidney and heart” according to *Integrating Chinese and Western Medicine*. JAK2/STAT3, PI3K/Akt, p38 MAPK, Wnt/β-catenin, GRP 78/PERK/CHOP, and ROS/GRP78/Caspase-12 signaling pathways are primarily regulated by ATL I and III, and exert cardio-renal protective effect, but there are no studies on the cardiac and renal protective effects of ATL II.

##### Cardiac and Renal Protective Effects of Atractylenolide I

Many major cardiac illnesses, such as myocardial infarction, are caused by myocardial ischemia/reperfusion (I/R) injury. ATL I was discovered in both in vitro and in vivo studies to minimize the level of damage, decrease apoptosis, and inhibit oxidative stress in cardiac I/R-injured cells through mechanisms that may be related to mitochondrial function protection and Caspase-3 inhibition [81]. By targeting FLT3 protein and inhibiting FLT3 phosphorylation, ATL I can inhibit the activation of PI3K/AKT pathway, reduce the production of HIF1-α, and inhibit the osteogenic differentiation of VIC, which has the effect of treating calcified aortic valve disease [82]. Guo et al. [83] discovered that ATL I inhibited renal fibrosis and reduced cell proliferation in unilateral uretera obstruction (UUO) mice. The mechanism may be inhibition of proliferation-related cascades composed of JAK2/STAT3, PI3K/Akt, p38 MAPK, and Wnt/β-catenin pathways, and reduced targeting the fibroblast-myofibroblast differentiation (FMD) and epithelial-mesenchymal transition (EMT) by inhibiting TGF-β-Smad2/3 signaling cascade.

##### Cardiac and Renal Protective Effects of Atractylenolide III

Endoplasmic reticulum stress (ERS) and excessive ERS-induced apoptosis in cardiac myocytes are two factors that contribute to the development of chronic heart failure. ATL III was discovered to reduce apoptosis and ROS, MDA, and LDH levels in H_2_O_2_-induced endoplasmic reticulum stress-injured cardiomyocytes H9c2, increase SOD activity and protect cardiomyocytes by regulating the ROS/GRP78/Caspase-12 signaling pathway, which could be a potential drug for the treatment of chronic heart failure according to Zuo et al. [84]. In addition, by inhibiting GRP 78/PERK/CHOP signaling pathway, ATL III can also inhibit the endoplasmic reticulum stress injury induced by chlamycin and the apoptosis of H9c2 cardiomyocytes, thus protecting myocardium [85]. In patients with advanced chronic renal disease, muscle atrophy owing to oxidative stress is a typical consequence. It was discovered that ATL III improved the degree of skeletal muscle atrophy, reduced mitochondrial damage, increased antioxidant enzyme activity, thereby reducing the production of reactive oxygen species, and reduced oxidative stress and autophagy in rats with advanced chronic kidney disease muscle atrophy induced by oxidative stress, which may exert anti-muscle atrophy effect through activation of oxidative stress-mediated PI3K/Akt/mTOR pathway [86].

#### 3.3.3. Hepatoprotective Effects

TLR4/MAPKs/NF-κB, AMPK/SIRT1, and cGAS/IL-1α/NF-κB signaling pathways are primarily regulated by ATL I and III, which provide hepatoprotective effects, but there are no studies on the hepatoprotective effects of ATL II.

##### Hepatoprotective Effects of Atractylenolide I

ATL I relieves hepatotoxicity, down-regulated plasma ALT and AST levels, reduces MDA, GSH, CAT activity and production of IL-1β, IL-6, and TNF-α, and inhibits TLR4/MAPKs/NF-κB signaling in a mice model of acetaminophen-induced acute liver injury [87]. Wang et al. [88] used BCG combined with lipopolysaccharide-induced immune liver injury mice to show that ATL I could alleviate liver injury, reduce the inflammatory response to liver injury, reduce MDA synthesis, and increase GSH-px activity, all of which could produce anti-acute liver injury by inhibiting the release or overexpression of inflammatory mediators such as NO, TNF-α, and iNOS.

##### Hepatoprotective Effects of Atractylenolide III

ATL III improved hepatic steatosis and reduced hepatic inflammation, fibrosis, and oxidative stress in mice with high-fat-diet-induced NAFLD, and reduced lipid accumulation and ROS production in free fatty acid-treated HepG2 cells by activating the hepatic AdipoR1-mediated AMPK/SIRT1 signaling pathway [89]. In addition, ATL III can inhibit the production of ASCT2 in activated hepatic stellate cells aHSC by inhibiting the cGAS/IL-1α/NF-κB pathway so as to enhance the expression of SASP, promote the aging of aHSC, and produce the anti-fibrosis effect, which may be related to the OH group in ATL III [90,91].

#### 3.3.4. Lung Protection Effects

TLR4/NF-κB and Nrf2/NQO1/HO-1 signaling pathways are primarily regulated by ATL I and III, which exert anti-lung damage actions, but there are no studies on the lung protection effects of ATL II.

Zhang et al. [92] discovered that ATL I protected mice against LPS-induced acute lung injury by suppressing TLR4 expression and NF-κB activation, as well as reducing inflammatory cytokine production. Another study determined that ATL III up-regulated the expression of FoxO1 and VNN1 proteins, inhibited the production of inflammatory molecules, and enhanced lung function and apoptosis in mice with sepsis-induced lung damage [93]. Huai et al. [94] used bleomycin-induced pulmonary fibrosis model rats to discover that ATL III improved lung injury and function by promoting the Nrf2/NQO1/HO-1 pathway, decreasing Caspase-3, Caspase-9, TGF-β, and α-SMA expression, down-regulating IL-6, iNOS, and TNF-α levels, up-regulating IL-10 levels, and increasing SOD and GSH activity.

#### 3.3.5. Neuroprotective Effects

ATL I and III reduce oxidative stress damage, neuronal cells damage, neuronal inflammation and enhance brain cells activity to generate anti-dementia and anti-depression neurological benefits, while ATL III has a unique therapeutic effect on microglia inflammation, but there are no studies on the neuroprotective effects of ATL II.

##### Neuroprotective Effects of Atractylenolide I

Alzheimer’s disease may be caused by a disruption of the blood–brain barrier. In human dopaminergic cells, ATL I was determined to reduce 1-methyl-4-phenylpyridine-induced SH-SY5Y pro-apoptotic protein levels, increase Bax/Bcl-2 levels, and enhance heme oxygenase protein production [95]. Gao et al. [96] determined that ATL I exhibited antidepressant effects in a chronic unpredictable mild stress (CUMS) mice model, and the mechanism may be related to the inhibition of NLRP3 inflammatory vesicle activation and hence reduction in IL-1β production.

##### Neuroprotective Effects of Atractylenolide III

ATL III has neuroprotective effects by reducing glutamate-induced neuronal death by partially blocking the Caspase signaling pathway [97]. Zhao et al. [98] discovered that ATL III can prevent homocysteine-induced apoptosis in primary cultured neurons by reducing Caspase-3 signaling, improve homocysteine-induced learning memory deficits, reduce ROS production, and prevent p-PKC expression decline in rats. ATL III suppresses isoflurane-induced apoptosis and autophagy in rat hippocampus neurons, lowers TNF-α, IL-6, and IL-1β production, and minimizes neurological damage through activating the PI3K/Akt/mTOR signaling pathway [99]. Furthermore, ATL III reduced cellular damage in corticosterone-injured rat PC12 cells by reducing intracellular Ca^2+^ excess, limiting mitochondrial apoptotic pathway, and modulating MAPK/NF-κB inflammatory pathway [100]. By reducing the degree of inflammation in hippocampus neurons, it may have antidepressant and anxiety-like effects in LPS-induced and CUMS model rats [101], which has been shown to be useful in the treatment of depression.

### 3.4. Regulate Blood Sugar and Blood Lipid

ATL I, II, and III primarily regulate blood glucose and lipid levels via influencing the AMPK and PI3K/Akt signaling pathways.

Chao et al. [102] discovered that by alleviating TNF-α-induced insulin resistance, increasing GLUT4 protein expression and activating the AMPK-activated protein kinase and PI3K/Akt signaling pathways, ATL I and II had a significant stimulatory effect on C2C12 myotubular glucose uptake. Another study demonstrated that by increasing AMPK phosphorylation, upregulating SIRT-1 and PGC1α protein expression, ATL III could boost glucose absorption in C2C12 myotubes and improve skeletal muscle energy metabolism, resulting in an anti-obesity and type 2 diabetes impact [103]. ATL II and III are bile acid receptor antagonists, and ATL III is also a progesterone agonist, according to Tsai et al. [104]. This research shows that ATL II and III could be used to treat hyperlipidemia and excessive vaginal bleeding. ATL III selectively decreased cholesterol [14C] oleate production in Chinese hamster ovary (CHO) K1 cells by limiting sterol O-acyltransferase (SOAT) activity, and has potential as an anti-hypercholesterolemic and anti-atherosclerotic medication [105].

### 3.5. Other Effects

ATL I, II, and III are effective in anti-platelet therapy, lowering blood pressure, and displays anti-bacterial and anti-viral, anti-radiation, hypothermia and anti-osteoporosis functions.

Most cardiovascular diseases, such as hypertension, stroke, atherosclerosis, and arterial thrombosis, are caused by impaired or altered platelet activity [106]. In mice with iron chloride-induced carotid artery thrombosis, ATL II and III not only reduced agonist-induced platelet aggregation and ATP secretion, downregulated p-Akt and p-p38 MAPK levels, inhibited platelet proliferation and clot contraction, but also prolonged the time to first occlusion and prolonged bleeding [107]. Disseminated intravascular coagulation can be caused by platelet aggregation. Tang et al. [108] discovered that ATL I could prevent lipopolysaccharide-induced diffuse intravascular coagulation and inhibit TNF-α levels via blocking the NF-κB signaling pathway.

ATL I and III are hypotensive and useful in the treatment of aldosteronism [109] and pre-eclampsia. Pre-eclampsia is a common hypertension disease that increases the risk of maternal and fetal morbidity and mortality during pregnancy [110]. ATL III improved HTR-8/SVneo viability and migratory ability of human extravillous trophoblast cells in early pregnancy and decreased H_2_O_2_-induced oxidative stress and apoptosis, according to Liu et al. [111], suggesting that it could be used to treat pre-eclampsia.

Zhou et al. [112] investigated the anti-rotavirus effects of ATL I, II, and III and discovered that ATL III not only directly inactivated rotavirus, but also reduced the diarrheal symptoms of rotavirus-infected mammary rats, whereas ATL I and II did not. According to other researchers, ATL I and III can inhibit methicillin-resistant Staphylococcus aureus and have distinct antibacterial properties [113]. In addition, ATL I can also improve fungal keratitis by inhibiting the secretion of MyD88/NF-κB pathway and IL-1β and IL-10, and enhance the efficacy of natamycin [114].

ATL I also has been demonstrated to be effective in the treatment of cancer cachexia [115]. By inhibiting the STAT3/PKM2/SNAP23 pathway, ATL I reduces the production of IL-6 and extracellular vesicles in C26 tumor cells and can be used to treat cancer cachexia [116]. Research discovered that ATL II can reduce radiation damage on human immortalized keratinocytes HaCaT by regulating the p38 MAPK/Nrf2 signaling pathway and upregulating the expression of HO-1 and NQO-1. Animal studies also confirmed that ATL II can reduce the radiation injury caused by ionizing radiation in mice [117]. By activating SIRT1/PGC-1α pathway, ATL III can increase the expression of thermogenic protein BAT and iWAT, which can be used to treat hypothermia [118]. ATL I and III can promote cartilage differentiation of bone marrow mesenchymal stem cells (BMSC) [119]. Further research has discovered that ATL I and II have been proven to protect against bone loss by blocking osteoclast differentiation [120].

**Figure 5:**
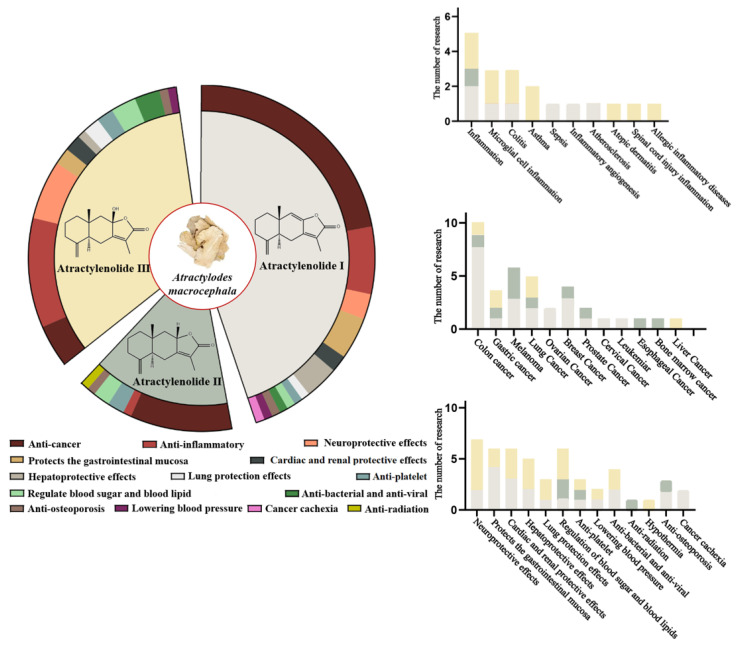
The number of researches on pharmacological effects of atractylenolides. Different color regions represent different types of effects, and the size of the color region represents the number of researches.

The number of researches on pharmacological effects of atractylenolide I, II, and III is shown in Figure 5.

## 4. Safety Toxicology

The safety of ATLs is a concern that must be addressed. However, no negative effects have been reported in clinical trials. According to the existing research data, ATLs can inhibit cell growth and have weak toxicity to cells. SW480 cells are mildly cytotoxic when administered 200 g/mL ATL I (10–15% inhibition) [41]. The cytotoxicity of ATL I, II, and III to Caco-2 was reported to be larger than 50, 70, and 480 mol/L, respectively [112]. Hepatotoxicity is a typical medication adverse effect usually produced by the combination of several pharmacological components. It has been demonstrated that ATL I enhances coumarin-induced hepatotoxicity. A total of 300 µM ATL I was cytotoxic to HepG2 cells when co-administered with 200 µM coumarin, but there was no further increase in toxicity when astragaloside IV was added [121]. Additionally, ATL I and III have strong specific inhibition of UDP-glucuronosyltransferase 2B7 (UGT2B7), which is one of the most important phases II drug-metabolizing enzymes and is involved in the metabolism of a variety of medicinal medications [122]. As a result, the effect of ATLs on metabolic enzymes may be a potential safety issue. Although it is unknown whether the toxic doses of ATLs mentioned above cause side effects in humans, it is interesting to note that ATL II has strong repellent activity against *T. castaneum* adults [123], ATL III has strong toxic effects against adult *Dermatophagoides farinae* and *Dermatophagoides pteronyssinus* [124], suggesting that it could be developed as a natural repellent or acaricide.

## 5. Clinical Research

At present, there is little research on the clinical application of ATLs, and further studies are needed. Liu et al. [125] separated 22 patients with malignant neoplasm of the stomach into observation and control groups, with the observation group receiving ATL I and the control group receiving fish oil-rich oral nutritional supplements. The observation group had a considerably better appetite, improved Karnofsky’s physical status, and a reduced PIF positive rate compared to the control group. Silicosis is a systemic disease induced by long-term inhalation of significant amounts of dust containing free silica during the manufacturing process, primarily due to pulmonary fibrosis [126]. Chen et al. [127] investigated 20 silicosis patients and discovered that ATL III suppressed autophagy and reduced apoptosis in silicosis alveolar macrophages by decreasing mTOR-dependent signaling pathways in cells without increasing lysosomal function, resulting in an anti-silicosis effect.

## 6. Discussion

*Atractylodes macrocephala* is a renowned warming tonic plant with an extensive history of use in clinical treatments. The primary characteristic and active components of this plant are ATL I, II, and III, which exhibit a range of pharmacological effects. Current research indicates that these three ATLs possess anti-cancer, anti-inflammatory, and organ-protective properties, among others, with differing activities and mechanisms. ATL I and II demonstrate potent anti-cancer effects, while ATL I and III exhibit more pronounced anti-inflammatory and organ-protective properties.

Anti-inflammatory activity is a crucial function of ATLs. Both ATL I and III inhibit the TLR4/NF-κB, PI3K/Akt, and MAPK inflammation-related pathways, thereby reducing the production of pro-inflammatory cytokines. Additionally, ATL III elicits anti-inflammatory effects by modulating the JAK2/STAT3, FPR1/Nrf2, and AMPK/SIRT1/PGC-1α signaling pathways. Conversely, research on ATL II’s anti-inflammatory properties is limited, suggesting that its efficacy in this regard is significantly lower than that of ATL I and III. ATL I and III have been determined to be effective in treating various inflammation-related diseases, such as asthma, spinal cord inflammation, and atherosclerosis, indicating that ATLs could be potential therapeutic agents for these conditions. To enhance their clinical value, further in vivo studies and clinical trials should be conducted to substantiate the therapeutic effects of ATL I, II, and III on inflammatory diseases.

The JAK2/STAT3 signaling pathway is a crucial target among the three ATLs pathways for cancer therapy. ATL I, II, and III function by inhibiting the activation of this pathway, which is achieved by impeding the phosphorylation of Jak3 and Stat3 as well as the nuclear translocation of Stat3. This inhibition leads to reduced cancer cell growth and migration while promoting apoptosis. Additionally, ATL I suppresses cellular glycolysis. ATL I and II modulate the cancer cell cycle through the PI3K/Akt and ERK/GSK3β signaling pathways, resulting in apoptosis and anti-tumor effects. Currently, research on the anti-cancer activity of ATL III is limited, while ATL I and II have been more extensively studied for their efficacy in treating colon, gastric, melanoma, lung, breast, and prostate cancers, with promising results. ATL I demonstrates strong effectiveness in ovarian cancer, and some studies suggest its potential in treating cervical cancer, bladder cancer, and leukemia, which have not been observed with the other two ATLs. Although ATLs have shown promise in various tumor types, many cancer types remain understudied, and the underlying mechanisms are not yet fully comprehended. Moreover, most of the research on ATL I, II, and III is focused on the cellular level, with a dearth of in vivo studies. To further elucidate the therapeutic potential of ATL I, II, and III in cancer treatment, it is imperative to strengthen in vivo research and conduct clinical trials.

ATLs exhibit protective effects on various organs, such as the heart, liver, lung, kidney, stomach, intestine, and nervous system. Specifically, ATL I and III contribute to organ protection by attenuating inflammation, modulating oxidative stress, activating anti-apoptotic signaling pathways, and inhibiting apoptosis. The organ-protective potential of ATL II is less documented, possibly due to its distinct structural properties. Consequently, ATLs hold promise as future multi-organ protective therapeutics with extensive development prospects.

Beyond their primary pharmacological effects, ATL I, II, and III possess several auxiliary benefits, such as anti-platelet activity, blood sugar and lipid regulation, and blood pressure reduction. However, given the limited scope of existing research and the many unidentified pathways, further in-depth investigations are necessary to unveil new activities for ATLs.

Moreover, under specific conditions, ATLs can interconvert, but distinct types of ATLs demonstrate varying activities and therapeutic effects on different diseases. As such, the stability of ATLs presents a potential challenge for their pharmacological application, warranting additional research to ensure consistent efficacy.

Despite the widespread use of *Atractylodes macrocephala* in traditional Chinese medicine, the safety of ATLs warrants attention. Although no adverse effects are reported clinically, studies have shown that ATLs exhibit cytotoxic properties and can exacerbate coumarin-induced hepatotoxicity. Additionally, due to their potent and selective inhibition of in vivo drug-metabolizing enzymes, ATLs may pose a safety risk. Therefore, further investigation of their safety profile is imperative.

## 7. Conclusions

ATL I, II, and III demonstrate promising anti-inflammatory, anti-cancer, and organ-protective properties. Future research should explore their therapeutic efficacy, pharmacological mechanisms, and clinical applications, as well as their safety, to facilitate broader clinical utilization.

## Figures and Tables

**Table 1:** Anti-inflammatory effect and mechanism of atractylenolide I, II, and III.

Inflammatory Disorders	Active Compound	Dosage	Models	Mechanism of Action	Ref.
Inflammation	I	1–100 μM	LPS-induced RAW264.7 cells	Suppresses MD-2, CD14, SR-A, TLR4 and MyD88 expression, reduces IL-6 and TNF-α production by inhibiting NF-κB, ERK1/2 and p38 signaling pathways	[21]
	II	3–100 μM	LPS-induced macrophages RAW264.7	Reduces NO secretion	[28]
	III	50–100 μM	LPS-induced macrophages RAW264.7	Inhibits ERK1/2, p38, and JNK1/2 expression, reduces NO, PGE2, TNF-α, and IL-6 production and through inhibition of NF-κB and MAPK signaling pathways	[29]
	I, III	1–100 μM	LPS-induced macrophages	Inhibits TNF-α expression and reduces NO production and iNOS activity	[22]
Microglial cell inflammation	I	25–100 μM; 3 mg/kg	LPS-induced inflammation in BV2 microglia; tetrahydropyridine poisoned mice	Reduces nuclear translocation of NF-κB and induction of HO-1	[23]
	III	100 μM	Immortalized mice microglia MG6	Inhibits p38 MAPK and JNK signaling pathways by down-regulation of TLR4 reduces TNF-α, IL-1β, IL-6, iNOS, and COX-2 production	[37]
	III	10 mg/kg	Mice model of transient middle cerebral artery occlusion	Reduces expression of pro-inflammatory factors IL-1β, TNF-α, IL-6, and anti-inflammatory factors by inhibiting JAK2/STAT3/Drp1 signaling pathway	[38]
Colitis	I	50 mg/kg	DSS-induced colitis in mice	Inhibits PI3K-Akt signaling pathway through suppression of SPHK1 and B4GALT2 gene variants in colitis colon, reduction in TNF-α, IL-6, IL-1β secretion, down-regulation of MUC2 and ZO-1, ocludin expression, and regulation of intestinal flora	[27]
	III	10–50 mg/kg	TNBS-induced colitis in mice	Reduces inflammatory factors IL-1β and TNF-α expression, decreases MPO content, increases CAT, SOD, and GSH-Px, decreases MDA and ROS levels, and regulates intestinal flora by inhibiting FPR1 and Nrf2 signaling pathways	[31]
	III	40–80 μM; 5–10 mg/kg	Lipopolysaccharide-induced IEC-6 cells; DSS-induced ulcerative colitis in mice	Alleviates mitochondrial dysfunction by activating AMPK/SIRT1/PGC-1α signaling pathway	[32]
Asthma	III	100 μg/mL; 100 mg/mL	IL-4-induced human bronchial epithelial-like cells 16HBE; ovalbumin-induced asthma in mice	Inhibits Cleaved Caspase-1, ASC, and NLRP3 expression, reduces NLRP3 inflammatory vesicle activation, and regulates Th1/Th2 balance	[34]
	III	0.1–10 mg/kg	Ovalbumin-induced asthma in mice	Reduces inflammation and oxidative stress by inhibiting STAT3 expression in lung tissue	[33]
Sepsis	I	10–40 mg/kg	Cecum ligation puncture in septic mice	Reduces WBC, LPS, TNF-α, IL-1β, IL-6, ALT, AST, Cre, and BUN levels	[24]
Inhibition of inflammatory angiogenesis	I	5–20 mg/kg	Freund’s complete adjuvant-induced air sac mice model, mice aortic ring model co-cultured with abdominal macrophages	Reduces NO, TNF-α, IL-1β, IL-6, VEGF, and PlGF production	[25]
Atherosclerosis	I	12.5–100 μg/mL	Ox-LDL-induced vascular smooth muscle cells	Reduces inflammatory cytokine production and MCP-1 expression, and inhibits p38-MAPK and NF-κB activation	[26]
Atopic dermatitis	III	1–100 μM	TSLP-induced HMC-1 human mast cells	Decreases production of pro-inflammatory cytokines, decreased Bcl2 and proCaspase-3 levels and increased p53, Caspase-3, and cleaved PARP levels by downregulating pSTAT6 and MDM2 levels	[30]
Spinal cord injury inflammation	III	1–100 μM; 5 mg/kg	Lipopolysaccharide-induced inflammation in BV2 microglia; rats with spinal cord injury	Promotes cellular M1 to M2 conversion by inhibiting NF-κB, MAPK/JNK, p38 MAPK, and Akt signaling pathways, further inhibiting the expression of corresponding inflammatory factors	[36]
Allergic inflammatory diseases	III	4–200 μM	IgE/Ag-induced basophilic leukemia cells in rats	Reduces IL-4 and TNF-α levels, inhibits phosphorylation of Lyn, Fyn, Syk, LAT, PLCγ, Gab2, Akt, p38, and JNK kinases, and increases Ca^2+^ levels	[35]

**Table 2:** Anti-cancer effects and mechanism of atractylenolide I, II, and III.

Types of Cancers	Active Compound	Dosage	Models	Mechanism of Action	Ref
Colon cancer	I	100–200 μM	HT-29	Induces DNA fragmentation in cells, decreases Caspase 9, Caspase 3, Caspase 7, Caspase 8, and PRAP expression, down-regulates Bcl-2 expression, and up-regulates Bax, Bak, Bad, Bim, Bid, and Puma expression	[39]
	I	30–50 μM	HCT116 and MC38	Enhances MHC-I-mediated antigen presentation by binding to the target protein PSMD4 and increases cytotoxic responses of CD^8+^ T cells	[45]
	I	25–100 μg/mL; 25–50 mg/kg	HCT116 and SW480; azomethane- and DSS-induced colon cancer in mice	Inhibits Drp1-mediated mitochondrial division; inhibits NLRP3 inflammatory vesicle activation in colitis-associated colorectal cancer	[41]
	I	80–200 μM; 25–75 mg/kg	HCT116 and COLO205; xenograft colorectal cancer nude mice	Inhibits glucose metabolism and disrupts dry maintenance through inhibition of the Akt/mTOR signaling pathway	[42]
	I	100–200 μM; 50 mg/kg	HCT116 and SW480; xenograft colorectal cancer mice	Inhibits glycolysis in CRC cells through inhibition of the JAK2/STAT3 signaling pathway and reduction in HK2 expression	[44]
	I	200 μM	LoVo and HT29 colorectal cancer stem cells	Inhibits the function of colorectal cancer stem cells and blocks the transfer of oncogenic miR-200c by impeding the delivery of extracellular vesicle uptake, thereby inhibiting the PI3K/Akt/mTOR signaling pathway	[43]
	I	25–100 μM; 25–50 mg/kg	HCT116; APC^Min/+^ mice	Reduces intestinal adenoma formation by down-regulation of D-dopachrome isomerase through activation of autophagy	[46]
	I	100 μM	HCT116	Decreases PDK1 and inhibited FoxO1 phosphorylation	[40]
	II	12.5–200 μg/mL	SW480, HCT116, Lovo and SW620	Inhibits the LncRNA XIST/miR-30a-5p/ROR1 signaling pathway	[61]
	III	100–200 μM	HCT-116	Promotes Bax, Caspase-9, and Caspase-3 expression; inhibits Bcl-2 expression and regulates the Bax/Bcl-2 apoptotic signaling pathway	[69]
Gastric cancer	I	25–100 μM	MGC-803	Inhibits proliferation through inhibition of the Notch signaling pathway	[56]
	II	200–400 μM	HGC-27 and AGS	Up-regulates Bax expression and down-regulates Bcl-2 expression through inhibition of Ras/ERK and PI3K/Akt signaling pathways	[62]
	III	50 μM	AGS and SGC-7901	Inhibits FGFR1, FGFR2, and FGFR4 expression	[73]
	III	80–120 μM; 1.2–2.4 mg/kg	AGS and HGC-27;MNNG-induced gastric precancerous lesions in rats	Inhibits HIF-1α and VEGF-A related to angiogenesis and down-regulation of DLL4	[74]
Lung cancer	I	50–150 μM; 25–75 mg/kg	A549 and H1299; A549 xenograft lung cancer nude mice	Inhibits PDK1 expression by activating ERK1/2 to suppress SP1 levels and reducing Stat3 levels	[49]
	I	10–40 μM	A549 and HCC827; A549 xenograft lung cancer nude mice	Up-regulates Caspase-3, Caspase-9, and Bax, down-regulates Bcl-2 and Bcl-xL	[50]
	II	2.5–5 μM; 50 mg/kg	A549; xenograft lung cancer nude mice	Inhibits M2-like polarization	[66]
	III	10–100 μM	A549	Causes cleavage of PAPR by enhancing Caspase-3 and Caspase-9 protein expression, and activating the mitochondrial pathway	[70]
	III	8–32 μM	LLC, H1703, H520, PC-9, A549 and H1299; LCC xenograft lung cancer nude mice	Down-regulates interferon-γ-induced IDO expression in lung cancer cells and activates anti-tumor immunity through inhibition of Jak3 and Stat3 phosphorylation and nuclear translocation of Stat3	[72]
Melanoma	I	100 μM	B16	Increases p21, Caspase 3, Caspase 8, and p53 expression and decreases CDK2, p-ERK, p-GSK3β, and c-Jun expression through inhibition of ERK/GSK3β signaling pathway	[51]
	I	40–150 μM	A375	Down-regulates STAT3 target genes Bcl-xL, MMP-2, and MMP-9 levels through inhibition of the JAK2/STAT3 signaling pathway	[52]
	II	100 mM	B16	Up-regulates p38, p53, p21, p27, Caspase-8, Caspase-9, and Caspase-3, down-regulates CDK2, p-Akt, p-ERK and Bcl-2	[63]
	II	20–40 μM; 12.5–25 mg/kg	B16 and A375; Melanoma cell B16 xenografts in mice	Reduces p-STAT3, p-Src, Mcl-1, and Bcl-xL through inhibition of STAT3 signaling pathway	[64]
	I, II	25–100 μM	B16	Inhibits Ras/ERK and PI3K/Akt signaling pathways	[65]
Ovarian cancer	I	50–100 μM	A2780	Up-regulates Bax, cleaved Caspase-9, cleaved Caspase-3, cytochrome c, and AIF expression, down-regulates B1, CDK1, and Bcl-2 expression, and inhibits PI3K/Akt/mTOR signaling pathway	[47]
	I	100 μM	SKOV3 EOC	Induces immunosuppressive molecules and products immunosuppressive T cells through activation of the MyD88/NF-κB signaling pathway	[48]
Breast cancer	I	50–100 μM; 50 mg/kg	MDA-MB-231 and HS578T; TNBC, breast cancer cells MDA-MB-231 xenografts in mice	Sensitizes cells to paclitaxel by blocking CTGF expression and fibroblast activation	[53]
	I	20–50 μM	MDA-MB-231	Glycolysis/gluconeogenesis is affected by down-regulating the expression of TPI 1 and GPI	[54]
	I	50–100 μM; 100–200 mg/kg	MCF 10A, MCF-7 and MDA-MB-231; N-Nitroso-N-methylurea-induced breast cancer in rats	Reduces NF-κB-regulated cytokines in breast cancer cells by inhibiting the TLR4/NF-κB signaling pathway	[55]
	II	50 μM; 100–200 mg/kg	MCF 10A; N-Nitroso-N-methylurea-induced breast cancer in rats	Elevates Nrf2 expression, nuclear translocation and expression of its downstream detoxification enzymes through the JNK/ERK-Nrf2-ARE signaling pathway; reduces inflammation and oxidative stress	[67]
Bladder Cancer	I	10–30 μM; 25–75 mg/kg	RT4, 5637, 253J and T-24; Xenograft bladder cancer nude mice	Inhibits PI3K/Akt/mTOR signaling pathway, up-regulates p21 expression and down-regulation of cyclin B1, CDK1, and Cdc25c expression	[58]
Leukemia	I	50–100 μg/mL	K562 (CML), U937 (AML) and Jurkat T	Up-regulates Caspase-3 and Caspase-9 expression, down-regulates proCaspase-3 and proCaspase-9 expression, and elevates CD14 and CD14/CD68	[57]
Cervical cancer	I	20–80 μM	Hela and SiHa	Combinates therapy with the P2X7R receptor antagonist JNJ; enhances cell growth inhibition	[60]
Prostate cancer	I	10 μM	DU 145 and PC-3	Inhibits Hsp27 expression	[59]
	II	50–100 μM	DU145 and LNCaP	Inhibits AR expression and activation of PIAS1 and JAK2/STAT3 signaling pathway	[68]
Liver cancer	III	10–500 μM	HepG2 and SMMC7721	Up-regulates miR-195-5p expression and down-regulates FGFR1 expression	[71]

## Data Availability

All date that support the findings of this study are include within the article.

## Abbreviations

ATLs	Atractylenolides
LPS	Lipopolysaccharide
FCA	Freund’s complete adjuvant
DSS	Dextran sulfate sodium salt
TSLP	Thymic stromal lymphopoietin
TNBS	Trinitrobrnzen sulfonic acid
AOM	Azoxymethane
DGP	Diabetic gastroparesis
I/R	Ischemia/Reperfusion
UUO	Unilateral uretera obstruction
ERS	Endoplasmic reticulum stress
CUMS	Chronic unpredictable mild stress
CHO	Chinese hamster ovary
BMSC	Bone marrow mesenchymal stem cells
SOAT	Sterol O-acyltransferase
UGT2B7	UDP-glucuronosyltransferase 2B7
